# Evaluating Standard of Care and Obstetrical Outcomes in a Reduced Contact Prenatal Care Model in the COVID-19 Pandemic

**DOI:** 10.1007/s10995-023-03812-3

**Published:** 2023-11-14

**Authors:** Jenny Y. Mei, Megan E. Bernstein, Eden Patton, Hai-Lang Duong, Masaru Negi

**Affiliations:** 1 Division of Maternal-Fetal Medicine, Department of Obstetrics and Gynecology, University of California, Los Angeles, CA USA; 2 David Geffen School of Medicine, University of California, Los Angeles, CA USA; 3 Division of Maternal-Fetal Medicine, Department of Obstetrics and Gynecology, Olive-View Medical Center, University of California, 14445 Olive View Dr, Sylmar, Los Angeles, CA USA; 4 Shenandoah Valley Maternal Fetal Medicine, Valley Health, Winchester, VA USA

**Keywords:** Reduced contact prenatal care, Standard of care, Pregnancy outcomes

## Abstract

**Introduction:**

We aimed to investigate the impact of reduced contact prenatal care necessitated by the COVID-19 pandemic on meeting standards of care and perinatal outcomes.

**Methods:**

This was a retrospective case-control study of patients in low-risk obstetrics clinic at a tertiary care county facility serving solely publicly insured patients comparing reduced in-person prenatal care (R) over 12 weeks with a control group (C) receiving traditional prenatal care who delivered prior.

**Results:**

Total 90 patients in reduced contact (R) cohort were matched with controls (C). There were similar rates of standard prenatal care metrics between groups. Gestational age (GA) of anatomy ultrasound was later in R (p = 0.017). Triage visits and missed appointments were similar, though total number of visits (in-person and telehealth) was higher in R (p = 0.043). R group had higher GA at delivery (p = 0.001). Composite neonatal morbidity and length of stay were lower in R (p = 0.017, p = 0.048). Maternal and neonatal outcomes did not otherwise differ between groups. Using Kotelchuck Adequacy of Prenatal Care Utilization index, R had higher rates of adequate prenatal care (45.6% R vs. 24.4% C, p = 0.005).

**Discussion:**

Our study demonstrates the non-inferiority of a hybrid, reduced schedule prenatal schedule to traditional prenatal scheduling. In a reduced contact prenatal care model, more patients met criteria for adequate prenatal care, likely due to higher attendance of telehealth visits. These findings raise the question of revising the prenatal care model to mitigate disparities in disadvantaged populations.

**Supplementary Information:**

The online version contains supplementary material available at 10.1007/s10995-023-03812-3.

## Significance

Recent studies have explored the impact of reduced prenatal care models on obstetrical outcomes with overall mixed findings. We aimed to investigate the impact of a reduced contact prenatal care model necessitated by the COVID-19 pandemic on meeting standards of care and obstetric outcomes. We found that in a reduced contact prenatal care model, more patients obtained adequate prenatal care, standards of care were met, and obstetrical outcomes were similar to that of controls. These findings raise the question of raising the question of pursuing a revised prenatal care model in the future.

**Supplementary Information:**

The online version contains supplementary material available at 10.1007/s10995-023-03812-3.

## Introduction

Prenatal care was initially proposed in the early 1900s to address low infant birth weight and high maternal mortality rates and has become one of the most frequently used health services in the United States (Ballantyne, 1901a; Ballantyne, 1901b). In 2001, there were approximately 50 million prenatal visits, with a median 12.3 visits per pregnancy (Cunningham et al., 2018).

Studies have shown that use of prenatal care is associated with decreased maternal mortality, preterm birth, neonatal death, and stillbirth (Xu et al., 2010; Ozimek and Kilpatrick, 2018; Berg et al., 2010; Leveno et al., 2009). Larger studies of the value of prenatal care are limited by the information in large administrative datasets, the unclear role of selection bias in that those who adequately use prenatal care may be healthier at baseline, and the use of non-comparable, historical controls. Studies in recent years have investigated impact on obstetrical outcomes with reduced prenatal care models with overall mixed findings (Villar et al., 2001; Dowswell et al., 2015).

Disparities in adequate access to prenatal care have been stark, with 23% of non-Hispanic Black women and 18% of Hispanic women receiving inadequate prenatal care in 2016, compared with 11% of non-Hispanic white women (Osterman and Martin, 2016). While there are national platforms aimed at narrowing these gaps in access to care in vulnerable populations, such disparities continue and are still at risk of being exacerbated by events such as the COVID-19 pandemic (Onwuzurike et al., 2020).

Currently, traditional prenatal care in the United States consists of visits at 4-week intervals until 28 weeks, then every 2 weeks until 36 weeks, and weekly thereafter. The COVID-19 pandemic forced many obstetrical clinics to significantly reduce the number of in-person prenatal visits to reduce disease transmission risk (Boelig et al., 2020). We aimed to investigate the impact of a reduced contact prenatal care model necessitated by the COVID-19 pandemic on meeting standards of care and maternal and neonatal outcomes.

## Methods

We performed a retrospective case-control study of patients in a low-risk obstetrics clinic at a tertiary care county facility comparing a reduced in-person prenatal care cohort (R) over a 12-week period from March 16, 2020 to June 5, 2020 with a control group (C) receiving traditional prenatal care who delivered before March 16, 2020. The facility served exclusively patients under a public health insurance plan, which traditionally is an underserved and vulnerable population in the United States. All patients with multiple gestations, major fetal anomalies, and major preexisting medical conditions are automatically categorized into our high-risk obstetrics clinic and thus excluded from this study population.

The reduced contact prenatal care model was based on an established hospital-wide algorithm (Supplementary Table) limiting total number of in-person visits to five for those initiating prenatal care in the model. All other visits were scheduled as telehealth visits as deemed clinically necessary.

Primary outcome was meeting standard of care for prenatal care metrics. Standards of care criteria included gestational age (GA) at first prenatal visit, dating ultrasound, and anatomy ultrasound; number of triage visits; total number of ultrasounds, visits, and missed appointments; whether Pap smear screening, genetic screening, gestational diabetes screening, group B streptococcus screening, and Tdap administration were given; and the rate of postpartum readmissions.

Secondary outcomes included maternal and fetal or neonatal outcomes. Maternal outcomes included gestational weight gain, gestational age at delivery, preterm delivery, cesarean delivery, antepartum admission, preeclampsia, received IV magnesium, chorioamnionitis, shoulder dystocia, postpartum hemorrhage (EBL > 1000 mL), higher degree perineal laceration, and maternal length of hospital stay. Fetal and neonatal outcomes included rates of fetal growth restriction, macrosomia, 1- and 5-min APGARs, 5-min APGAR < 7, NICU admission, non-lethal anomaly, intrauterine fetal demise, and neonatal length of hospital stay. Composite neonatal morbidity was included and defined as any of the following being present: gestational age < 32 weeks, birthweight < 1500 g, neonatal death, respiratory distress syndrome, seizures, intraventricular hemorrhage, birth trauma, hypoxic ischemic encephalopathy, necrotizing enterocolitis, bronchopulmonary dysplasia, sepsis, pneumonia.

We also evaluated the adequate use of prenatal care between groups as defined by the widely used Kotelchuck Adequacy of Prenatal Care Utilization (APNCU) index, which has been found to be significantly associated with infant outcomes, particularly gestational age at delivery and birth weight (Osterman & Martin, 2018). The APNCU index modified the previously used Kessner index to define adequate prenatal care by month of care initiation and the expected, appropriate number of visits after initiation of care. Adequacy of received prenatal care services was calculated by observed number of prenatal visits divided by expected number of prenatal visits based on time of entry into prenatal care. Inadequate prenatal care was defined as this value being < 49%; intermediate if 50–79%; adequate if between 80 and 109%; and adequate plus if > = 110%.

Independent sample *t*-test, ANOVA, and Chi-square were used to compare outcomes between groups. SPSS version 21 (IBM SPSS Institute, Inc., Armonk, NY) was used to analyze data. For all analyses, p-values were two-sided, and the level of statistical significance was set at p < 0.05. This research was conducted in accord with prevailing ethical principles and reviewed by the Institutional Review Board of UCLA (IRB # 1629325-1).

## Results

A total of 90 patients met inclusion criteria in the reduced contact prenatal care (R) cohort of the pre-defined period and were matched with controls (C) who had also been in low-risk obstetrics clinic and without major co-morbidities. Demographics between the two groups are seen in Table 1. Maternal age was similar between groups (29.6 ± 6.3 years in R vs. 30.7 ± 6.2 in C, p = 0.26). Total in-person visits was lower in R cohort as compared to C (6.5 vs. 8.3, p < 0.001). Of note, while patients who initiated prenatal care in the R cohort were limited to 5 in-person visits, some patients in the overall cohort were partially through prenatal care when they entered the model, thus the average number of visits overall was slightly higher than 5. There was a lower percentage of nulliparous patients in the R cohort as compared to C (11.1% vs. 42.2%, p < 0.001). The racial demographics highlight the majority Hispanic patient population in our cohort, with over 50% in both cohorts. Other baseline demographics were similar between groups.

**Table 1:** Study population characteristics between reduced contact prenatal care cohort and controls

Demographic	Reduced contact prenatal care (n = 90)	Control (n = 90)	p-value^a^
Maternal age in years (mean ± SD)	29.6 ± 6.3	30.7 ± 6.2	0.26
Number of in-person visits	6.5 ± 2.5	8.3 ± 2.6	**< 0.001**
Nulliparous	10 (11.1%)	38 (42.2%)	**< 0.001**
Ethnicity			
Asian	3 (3.3%)	6 (6.7%)	
Black	7 (7.8%)	3 (3.3%)	
Hispanic/Latina	49 (54.4%)	59 (65.6%)	0.16
White	2 (2.2%)	8 (8.9%)	
None of the above	29 (32.2%)	14 (15.6%)	
BMI (kg/m^2^) at delivery (mean ± SD)	32.5 ± 6.8	33.4 ± 6.5	0.94
Prior preterm birth	7 (7.8%)	7 (7.8%)	1.0
Prior cesarean delivery	12 (13.3%)	17 (18.9%)	0.31

Bold values indicate p < 0.05

Data are represented as n (%) or mean ± standard deviation

*SD* standard deviation, *BMI* body mass index (kg/m^2^)

^a^P-values were calculated by *t*-test or Chi-square as appropriate

Standards of care metrics between the two cohorts is listed in Table 2. Gestational age (GA) of anatomy ultrasound (US) was later in R cohort (22 weeks vs. 20.8, p = 0.017). Number of triage visits and missed appointments were similar, though total number of visits (in-person and telehealth) was higher in R (9.2 vs. 8.3, p = 0.043). There were similar rates of standard prenatal care metrics. All Rh-negative patients received antepartum Rhogam.

**Table 2:** Standard of care criteria between reduced contact prenatal care cohort and controls

Standard of care	Reduced contact prenatal care (n = 90)	Control (n = 90)	p-value^a^
Gestational age of first prenatal visit (weeks)	16.1 ± 7.7	15.0 ± 6.6	0.31
Gestational age of dating US (weeks)	12.1 ± 6.5	11.0 ± 6.1	0.28
Gestational age of anatomy US (weeks)	22.0 ± 4.0	20.8 ± 2.8	**0.017**
Number of triage visits	1.3 ± 1.4	1.7 ± 2.1	0.19
Total number of ultrasounds	3.5 ± 1.7	3.6 ± 1.2	0.62
Total number of visits^b^	9.2 ± 2.8	8.3 ± 2.6	**0.043**
Total number of missed appointments	1.4 ± 1.8	1.0 ± 1.3	0.072
Pap smear screening	85 (94.4%)	87 (96.7%)	0.74
Genetic screening	73 (81.1%)	78 (86.7%)	0.42
Gestational diabetes screening	87 (96.7%)	88 (97.8%)	0.65
Tdap administration	88 (97.8%)	83 (92.2%)	0.09
Group B streptococcus screening	88 (97.8%)	89 (98.9%)	0.56
Postpartum readmission	6 (6.7%)	8 (8.9%)	0.58

Bold values indicate p < 0.05

Data are represented as n (%) or mean ± standard deviation

^a^*P*-values were calculated by *t*-test or Chi-square as appropriate

^b^Total visits as defined by in-person and telehealth clinic visits as deemed clinically necessary. This does not include triage visits

Obstetrical outcomes between the two cohorts are listed in Table 3. Gestational age (GA) at delivery was higher in the R group (39w2d vs. 38w3d, p = 0.001). Mode of delivery, preterm delivery, antepartum admission, preeclampsia, and postpartum hemorrhage did not differ between groups, nor did fetal growth restriction, NICU admission, and IUFD. Composite neonatal morbidity was lower in R (1.1% vs. 8.9%, p = 0.017), as was neonatal length of hospital stay (2.3 days vs. 4.4, p = 0.048).

**Table 3:** Obstetrical outcomes between reduced contact prenatal care cohort and controls

Obstetrical outcomes	Reduced contact prenatal care (n = 90)	Control (n = 90)	p-value^a^
Gestational weight gain (kg)	8.5 ± 6.2	8.7 ± 10.9	0.85
Gestational age at delivery	39w2d ± 8d	38w3d ± 13d	**0.001**
Preterm delivery	4 (4.4)	10 (11.1%)	0.09
Cesarean delivery	28 (31.1%)	33 (36.7%)	0.61
Spontaneous labor	46 (51.1%)	44 (48.9%)	0.27
Emergent cesarean delivery	3 (3.3%)	2 (2.2%)	0.65
Antepartum admission	11 (12.2%)	11 (12.2%)	1.0
Estimated blood loss (mL)	446.9 ± 344.8	491.4 ± 467.9	0.47
Blood transfusion	3 (3.3%)	2 (2.2%)	0.65
Preeclampsia	10 (11.1%)	15 (16.7%)	0.28
IV magnesium	6 (6.7%)	7 (7.8%)	0.77
Chorioamnionitis	3 (3.3%)	3 (3.3%)	1.0
Shoulder dystocia	0 (0.0%)	1 (1.1%)	0.32
Postpartum hemorrhage	5 (5.6%)	9 (10.0%)	0.27
Third- or fourth-degree laceration	4 (4.4%)	1 (1.1%)	0.17
Maternal length of stay (days)	3.5 ± 1.3	3.2 ± 3.4	0.43
Fetal/neonatal outcomes			
Fetal growth restriction	4 (4.4%)	9 (10.0%)	0.15
Fetal macrosomia	3 (3.3%)	6 (6.7%)	0.31
Neonatal birthweight (g)	3352 ± 509	3206 ± 609	0.08
1-min APGAR	7.9 ± 1.1	7.8 ± 1.4	0.96
5-min APGAR	8.8 ± 0.6	8.9 ± 0.4	0.22
5-min APGAR < 7	2 (2.2%)	0 (0%)	0.16
NICU admission	7 (7.8%)	15 (16.7)	0.07
Non-lethal anomaly	0 (0%)	1 (1.1%)	0.32
Intrauterine fetal demise	0 (0%)	3 (3.3%)	0.08
Composite morbidity	1 (1.1%)	8 (8.9%)	**0.017**
Neonatal length of stay (days)	2.3 ± 1.0	4.4 ± 10.1	**0.048**

Bold values indicate p < 0.05

Data are represented as n (%) or mean ± standard deviation

^a^*P*-values were calculated by *t*-test or Chi-square as appropriate

Using the Kotelchuck Adequacy of Prenatal Care Utilization (APNCU) index as seen in Table 4, the reduced contact prenatal care cohort had higher rates of patients who had adequate prenatal care (45.6% R vs. 24.4% C, p = 0.005), in which patients attended between 80 and 109% of their expected number of prenatal visits based on time of entry into prenatal care. The majority (63.3%) of patients in the control cohort had “intermediate” adequacy of prenatal care, with 50–79% attendance out of expected visits. Three patients in the R cohort had “adequate plus” prenatal care, with over 110% attendance; none of the patients in C cohort met these criteria.

**Table 4:** Adequacy of received prenatal care services

Adequacy of received prenatal care services^a^	Reduced contact prenatal care (n = 90)	Control (n = 90)	P-value^b^
Inadequate	7 (7.8%)	11 (12.2%)	**0.005**
Intermediate	39 (43.3%)	57 (63.3%)	
Adequate	41 (45.6%)	22 (24.4%)	
Adequate plus	3 (3.3%)	0 (0%)	

Bold value indicates p < 0.05

Data are represented as n (%) or mean ± standard deviation

^a^Adequacy of received prenatal care services as calculated by observed number of prenatal visits divided by expected number of prenatal visits based on time of entry into prenatal care. 0–49% = inadequate; 50–79% = intermediate; 80–109% = adequate; 110+% = adequate plus

^b^*P*-values were calculated by Chi-square as appropriate

## Discussion

Our study demonstrates the non-inferiority of a hybrid, reduced schedule prenatal schedule to traditional prenatal scheduling. Furthermore, in a reduced contact prenatal care model, more patients met criteria for adequate prenatal care as compared to controls.

Since its implementation over a century ago, increasing use of prenatal care has been shown to decrease the maternal mortality rate, preterm births, and stillbirths (Leveno et al., 2009; Vintzileos et al., 2002). However, 6 to 7% of American women have late or no prenatal care, a reality that disproportionately affects Hispanic and African American patient populations (Child Trends et al., 2015). This has ramifications for both maternal and fetal or neonatal outcomes. The Pregnancy Mortality Surveillance System identified a fivefold increased risk for maternal death in women who received no prenatal care (Berg et al., 2010). The risk for preterm birth, stillbirth, early and late neonatal death, and infant death rises linearly with decreasing prenatal care (Partridge et al., 2012).

In an analysis of birth certificate data, the Center for Disease Control found that half of women with delayed or no prenatal care wanted to begin care earlier, and the most cited reasons for lack of prenatal care were late recognition of pregnancy, lack of money or insurance, and inability to obtain an appointment (Cunningham et al., 2018). These issues were all thought to be potentially exacerbated by the COVID-19 pandemic.

Conversely, our reduced contact prenatal care cohort affected by the pandemic showed significantly higher rates of patients deemed to have adequate prenatal care per the APNCU index as compared to the control cohort. Furthermore, patients in the reduced contact prenatal care cohort had higher total number of visits (including both in-person and telehealth) as compared to controls. These findings are potentially due to the fact that underserved patient populations traditionally have higher rates of missed appointments in routine prenatal care (and medical care in general) due to difficulty with transportation, financial hardships, job insecurity, inability to take time off work, and other social determinants of health that disproportionately affect these populations and widen disparities in access to care (Gadson et al., 2017). With the replacement of telehealth in lieu of in-person visits, this cohort was able to attend more prenatal visits and may have had overall improved access to care. While some may raise concern about the impact on meeting standards of care with reduced in-person visits, we found no differences in meeting standard of care metrics between the two groups.

Previously, telehealth had mainly been used to deliver other ancillary services, such as tobacco cessation or nutrition counseling, with mixed results in obstetric patient populations (DeNicola et al., 2020). Duryea et al. recently found that a pregnant patient population who delivered in 2020 following implementation of audio-only prenatal virtual visits due to the pandemic did not experience more adverse perinatal outcomes compared with women who delivered in 2019 (Duryea et al., 2021). Similarly, Stowe et al. reported no increased rates of stillbirth during the initial 2 months of the COVID-19 pandemic lockdown in England, assuming pregnant women may have received fewer services or been hesitant to access healthcare during the pandemic (Stowe et al., 2020). Our findings echo these studies, showing no differences in perinatal outcomes between our reduced contact prenatal care cohort and our control cohort.

Previous international studies in the 2000s compared reduced prenatal visits to traditional models of care with mixed results. Villar et al. performed a multicenter randomized controlled trial through the World Health Organization with almost 25,000 women comparing routine prenatal care which required a median of 8 visits with a new model requiring a median of 5 and found that provision of care by the new model did not affect maternal and perinatal outcomes (Villar et al., 2001). These findings were corroborated by McDuffie et al. in a RCT comparing an experimental schedule of 9 visits with a control schedule of 14 that found no differences in perinatal outcomes or patient satisfaction (McDuffie et al., 1996). Sikorski et al. also showed similar perinatal outcomes, however, they did note lower patient satisfaction and poor psychological outcomes (Sikorski et al., 1996).

A Cochrane Systematic Review in 2010 subsequently combined all the randomized trials comparing reduced number of antenatal visits with standard care and found that in settings with limited resources where the number of visits is already low, reduced visits was associated with increased perinatal mortality (Dowswell et al., 2015). In response to these findings, a secondary analysis was done of the original Villar et al. WHO data that stratified women by baseline risk for and timing of perinatal death. They found that it is plausible that increased risk of fetal death could be due to reduced number of visits, though study heterogeneity and differences in quality of care and visit timing could affect outcomes (Voge et al., 2013). Both studies concluded that monitoring maternal, fetal, and neonatal outcomes when implementing antenatal care protocols is essential. Our study takes another look at the potential of implementing a reduced contact prenatal care model as necessitated by the COVID-19 pandemic and its possible effect on meeting standards of care as well as maternal, fetal, and neonatal outcomes.

A notable finding in our study is that in our underserved patient population, the patient cohort that had more telehealth than in-person visits had higher rates of utilization of prenatal care. Our results suggest that in the appropriate patient population, notably the underserved and vulnerable populations, providing a routine telehealth component may help improve overall access to care. These changes may help reduce disparities in care in these traditionally disadvantaged patient populations.

Strengths of our study include the comparison to controls rendering validity to the results. We focused on an underserved, vulnerable patient population in hopes of highlighting the existing disparities in prenatal care and elucidating ways of mitigating the impact from the COVID-19 pandemic on these communities.

The study is limited by its retrospective nature, although our method of including all deliveries in a linear temporal fashion from the initial onset of prenatal care changes and selecting a control cohort immediately prior to these changes may help reduce potential selection bias in our primary analysis. There were higher rates of nulliparous patients in the control group, which could affect obstetrical outcome, though we did not find significant differences in maternal outcomes between groups. There may be potential confounders from the COVID-19 pandemic that affected certain metrics, such as patients feeling less comfortable with coming to the hospital or having financial difficulties that impacted their ability to access care. Some patients may even have had less time restraints to seek medical care while unemployed or furloughed. Furthermore, only low risk obstetrics clinic patients were included in this analysis, thus our findings may not be generalizable to patients with comorbidities or complications during pregnancy. Of note, our study was done in a single tertiary-care center that serves an underserved population but is in a high resource country and thus may not be generalizable to certain international settings. Patients should be risk-stratified to appropriate degrees of in-person versus virtual prenatal care; the delivery of prenatal care should be tailored to the patient.

Next steps include evaluating patient satisfaction between the two cohorts of reduced in-person prenatal care and standard prenatal care. While this has been studied in the past with mixed results, this may be different in an underserved patient population that has limited access to in-person care (Villar et al., 2001; Dowswell et al., 2015). Butler Tobah et al. found that a reduced-frequency prenatal care model resulted in higher patient satisfaction and lower prenatal stress as reported by patients; of note they did not measure obstetric outcomes (Butler Tobah et al., 2019). Another recent study done during the COVID-19 pandemic reported improved patient satisfaction with audio-only virtual care as well (Holcomb et al., 2020).

There was a higher total number of visits in the reduced contact prenatal care cohort, and it is possible this cohort may have higher satisfaction. Future research necessitates large scale prospective studies with sufficient power to detect meaningful differences in pregnancy outcomes and subsequently investigate the impact of a reduced contact prenatal care model outside of the COVID-19 pandemic to negate any confounders.

In a reduced contact prenatal care model, standards of care are met, and obstetrical outcomes are similar to a standard prenatal care model. Interestingly, more patients in the reduced contact prenatal care cohort met criteria for adequate prenatal care as compared to controls, which may be attributable to higher attendance of telehealth visits as compared to in-person in a disadvantaged patient population. These findings raise the question of pursuing a revised prenatal care model outside of COVID-19 pandemic in the future, especially to help mitigate disparities in disadvantaged patient populations.

### Supplementary Information

Below is the link to the electronic supplementary material.

Supplementary material 1 (DOCX 13.9 kb)

## Data Availability

Not applicable.
