# Supplementary material for: Evaluating Standard of Care and Obstetrical Outcomes in a Reduced Contact Prenatal Care Model in the COVID-19 Pandemic
Source: Matern Child Health J. 2023 Nov 14;28(2):287–93. doi: 10.1007/s10995-023-03812-3 (PMC10901916; doi:10.1007/s10995-023-03812-3)
Supplement: Supplementary file 1 — Supplementary material 1 (DOCX 13.9 kb) [file 10995_2023_3812_MOESM1_ESM.docx]

Supplementary Table: Modified Prenatal Care Model

|  | **Phone Visit** | **Face-to-Face (FTF) Visit** |
| --- | --- | --- |
| **1^st^ trimester (10-12 weeks)** | | |
| New visit | If known LMP and prefers to defer screening to 18-20 week visit | If known LMP and desires 1^st^ trimester screening |
|  | If unknown LMP:  1. Ultrasound for CRL by Radiology  2. Prenatal labs | If unknown LMP:  1. Ultrasound for CRL by OB  2. Prenatal labs |
| **2^nd^ Trimesters (18-22 weeks)** | | |
| 18-19 week visit | Reminder to complete 2^nd^ trimester screening |  |
| 20-22 week visit |  | Anatomy US |
| **3^rd^ trimester (27-40 weeks)** | | |
| 27-30 week visit |  | 1. Labs (CBC, RPR, HIV, 1hr GLT)  2. Offer Tdap vaccine |
| 32-34 week visit | Phone visit if…  1. Low risk  2. Has home BP monitor  3. Reports normal weight gain  4. Reports fetal movement | FTF visit if…  1. High risk  2. Needs BP monitoring  3. Obesity (32 week growth scan) |
| 36-40 week visit | Biweekly phone visits if…  1. Low risk  2. Has home BP monitor  3. Reports normal weight gain  4. Reports fetal movement | FTF visits if…  1. GBS swab  2. High risk  3. Needs BP monitoring  4. Obesity - 32 week growth |

Abbreviations: LMP, last menstrual period; CRL, crown-rump length; OB, obstetrician; US, ultrasound; BP, blood pressure; GBS, group B streptococcus
